# Efficacy of 1064 nm Fractional Q‐Switched Laser and Topical Isotretinoin: A Split‐Face Randomized Clinical Trial

**DOI:** 10.1111/jocd.71017

**Published:** 2026-07-31

**Authors:** Gita Faghihi, Maryam Aghaei, Hamid Varastehpour, Sarah Seyedyousefi, Bahareh Abtahi‐Naeini

**Affiliations:** ^1^ Skin Diseases and Leishmaniasis Research Center, Department of Dermatology Isfahan University of Medical Sciences Isfahan Iran

**Keywords:** 1064 nm fractional Q‐switched laser, acne scars, topical isotretinoin

## Abstract

**Introduction:**

Acne scars are a common complaint amongst dermatology patients and treating these scars effectively can still be challenging. Multiple treatment approaches can be used on this matter, one of which is using various types of lasers. On the other hand, one of the most effective medical treatments for acne is topical retinoids, which can improve acne and prevent scarring. This study aimed to compare the combined effect of 1064 nm fractional Q‐Switched Laser and topical isotretinoin with 1064 nm fractional Q‐Switched Laser alone in the treatment of acne scars.

**Method:**

In this randomized clinical trial, 20 patients with bilateral acne scars were treated with Q‐Switched laser on one side of the face and Q‐Switched laser combined with topical isotretinoin on the other. Treatments were performed every 2 weeks for a total of three sessions. Scar improvement was assessed at 3 and 6 months by a blinded assessor concerning clinical features and using dermoscopic images by Fotofinder dermoscope. Statistical analyses were performed using repeated measures ANOVA.

**Results:**

Twenty patients (16 female and 4 male) with a mean age of 36.55 finished the study. The side treated with the combination therapy showed significantly better scar scores and percent change at both 3 and 6 months compared to the side treated with laser alone treatment (39.0 ± 2.60 vs. 23.9 ± 1.90; *p*‐value < 0.001 after 6 months of treatment). No severe adverse effects were observed. Only one patient experienced transient erythema.

**Conclusion:**

The combination of topical isotretinoin and Fractional Q‐Switched 1064 nm laser was more effective than the laser alone in reducing acne scars, with minimal side effects.

## 1 Introduction

Scarring is a common identifiable sequela of acne that negatively affects the individual's appearance and psychological state [1]. Acne scarring is a frequent and often persistent sequela of acne vulgaris, particularly in patients with inflammatory lesions, and may present as atrophic, hypertrophic, or keloidal scars. Management of atrophic post‐acne scars is challenging and usually requires procedural or combination approaches, including fractional ablative and non‐ablative lasers, microneedling with or without radiofrequency, chemical reconstruction techniques, subcision, fillers, and other resurfacing modalities. Recent evidence also suggests that topical retinoids may contribute to the prevention and improvement of atrophic acne scars through modulation of inflammation and dermal remodeling [2, 3, 4, 5, 6, 7].

Acne scarring is a frequent and often permanent sequela of acne vulgaris. Epidemiological studies suggest that approximately 30%–95% of patients with acne develop some degree of scarring, with moderate to severe scarring reported in up to 20%–30% of affected individuals. The presence of acne scars has been consistently associated with impaired quality of life, psychological distress, anxiety, and depression, underscoring the need for effective and safe therapeutic strategies [1].

{Citation}Isotretinoin, a retinoid derived from vitamin A, is considered the most effective pharmacological treatment for moderate to severe forms of acne with nodular and cystic manifestations due to its ability to target all major pathogenic factors of the disease. In addition to reducing sebaceous gland size and sebum production, isotretinoin modulates keratinocyte differentiation, exhibits anti‐inflammatory properties, and downregulates Toll‐like receptor 2 (TLR2) expression in monocytes, which plays a role in the innate immune response to Cutibacterium acnes. This drug reduces hyperkeratosis and indirectly inhibits the growth and proliferation of *Propionibacterium acnes* and increases collagen synthesis and improves skin [8]. Topical isotretinoin (13‐cis‐retinoic acid) has been studied for acne lesion control [9]. Recent evidence, however, suggests that certain non‐ablative lasers may be safely used alongside isotretinoin, potentially enhancing clinical outcomes without significantly increasing adverse effects [10]. This evolving understanding warrants further clinical investigation into combination therapies involving isotretinoin and fractional lasers.

Laser options for acne and acne scarring include ablative fractional systems (CO_2_, Er:YAG), non‐ablative fractional erbium‐glass (1540–1550 nm), and short‐pulse 1064‐nm Nd:YAG platforms (Q‐switched or picosecond), each targeting inflammation, sebaceous activity, and dermal remodeling to differing extents [11]. While ablative CO_2_ often achieves larger single‐session scar gains, it carries more downtime and post‐inflammatory hyperpigmentation risk, and head‐to‐head data show CO_2_ outperforming older Q‐switched 1064‐nm approaches on efficacy metrics [12]. In contrast, 1064‐nm fractional Q‐switched (QSF) Nd:YAG leverages micro‐fractionation and laser‐induced optical breakdown to stimulate neocollagenesis with shorter recovery and a favorable safety profile—particularly relevant for higher phototypes—with clinical studies showing meaningful scar improvement and minimal adverse effects [13]. Moreover, newer fractional 1064‐nm short‐pulse comparisons report comparable scar improvement to ablative fractional Er:YAG/CO_2_ but with fewer adverse effects and quicker recovery, underscoring a superior efficacy‐to‐downtime balance [14].

For combination regimens, accumulating evidence suggests fractional lasers can be used alongside isotretinoin without excess scarring or wound‐healing complications—and in some studies with better outcomes than delaying procedures—supporting the rationale for pairing QSF‐1064 nm with topical isotretinoin in our split‐face design [15, 16].

Given the favorable effects of isotretinoin in treating acne and preventing the formation of acne scars, limited studies that have been conducted recently have considered using isotretinoin and laser simultaneously and in combination to improve the disease, treat the scars caused by the disease, inhibit the process of scar formation as soon as possible, and increase the effectiveness of the laser. Considering this, we aim to compare the combined effect of 1064 nm fractional Q‐Switched Laser and topical isotretinoin with 1064 nm fractional Q‐Switched Laser alone in the treatment of acne scars.

## 2 Methods and Materials

### 2.1 Overview and Patients

A randomized assessor‐blinded clinical trial was conducted on 20 patients with acne scars.

Inclusion criteria included informed consent to participate in the study, age over 18 years, having almost identical scars on both sides of the face, Fitzpatrick skin phototype of 1–4, no history of keloid formation, no severe systemic diseases (e.g., uncontrolled diabetes) and no history of malignancy. The exclusion criteria were pregnancy or breastfeeding, history of hypersensitivity to laser and isotretinoin, and a history of using other medical treatments for their acne scars in the last 6 months.

The trial was conducted according to the Declaration of Helsinki and subsequent revisions and was registered at the Iranian of clinical trials (WWW.IRCT.IR; unique registration number: IRCT20110501006350N2). The written informed consent was previously obtained from all patients.

### 2.2 Study Protocol

This randomized controlled trial followed an assessor‐blinded design.

A study checklist was used to collect demographic data for each participant, including age, gender, family history, past medical history, and underlying medical conditions.

Twenty patients with bilateral almost symmetrical acne scars were enrolled in this study. A split‐face design was conducted with one side of the patient's face receiving only Q‐switched laser (Group 1) and the other side of the face receiving Q‐switched laser plus topical isotretinoin (Group 2). The allocation was completely random using random number generating and the responsible author for this randomization was not involved in the analysis of the study results.

The laser protocol involved three sessions, 2 weeks apart, using 1064 nm fractional Q‐Switched Laser with 2–4 J/cm^2^ fluence and two passes per area. Topical Isotretinoin 0.05% gel (Isoten, Raha pharmaceutical company, Iran) was applied once nightly for 30 min over a period of 3 months, starting from the initiation of treatment. Patients were advised not to take any other topical or systemic medications for the duration of the study. All participants were instructed by the investigator for appropriate use of topical isotretinoin.

Participants were instructed to avoid exfoliants and practice strict photoprotection using sunscreen starting day 1 after laser therapy and the start of the treatment protocol to mitigate inflammation.

### 2.3 Outcome Assessment

At baseline, after 3 and 6 months, two dermatologists who did not know which side of the face the intervention was performed, visited the patients. They scored the scars by goodman and baron's quantitative scar scale [17], on both sides of the face based on reviewing the images with the help of Photo finder and calculating acne scar score. At each visit all patients were assessed for possible complications.

### 2.4 Statistical Analysis

The categorical variables were reported as frequency (%), and continuous variables were reported as mean (SD). The unit of analysis for the split‐face was the side. In split‐face analysis, the trends and changes post‐treatment from the baseline measurements on each side were calculated. Comparisons were conducted using a two‐way repeated‐measures ANOVA, with Bonferroni adjustment for multiple comparisons. The mean differences between sides and the associated standard error were reported. A *p*‐value of < 0.05 was considered statistically significant. Analysis was performed using SPSS software (version 20).

## 3 Results

### 3.1 Baseline Characteristics

Twenty patients completed the study follow‐up. Patients' mean (SD) age was 36.55 (9.63) years with a range from 23 to 56, and 80% of the subjects were female.

### 3.2 Scar Score

**FIGURE 1:**
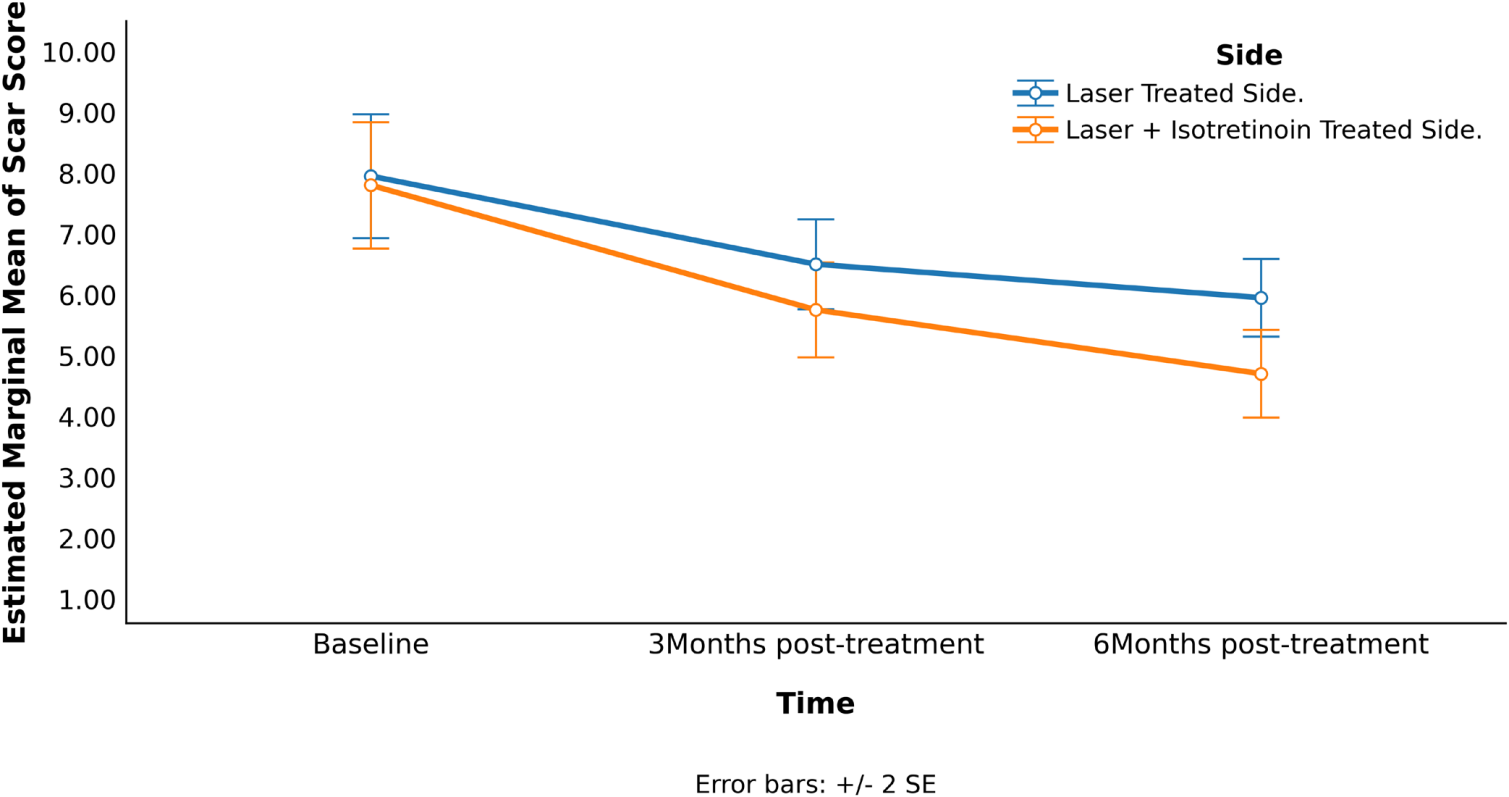
Scar score changes in both sides over study follow‐ups (mean ±2SE).

As shown in Table 1, a significant reduction was observed in the scar score on both sides of the face at 3 and 6 months after treatment (*p* < 0.001). The time × side interaction effect was statistically significant (*p* = 0.003), implying that the scar score reduced considerably sharply on the laser + isotretinoin‐treated side (Figure 1). Reduction in the scar score in the laser + isotretinoin‐treated side was significantly higher than the only laser‐treated side at 3 months (5.75 ± 0.39 vs. 6.50 ± 0.37; mean difference: −0.75 ± 0.190; *p*‐value < 0.001), and at 6 months (4.70 ± 0.36 vs. 5.95 ± 0.32; mean difference: −0.75 ± 0.190; *p*‐value < 0.001) after treatment.

**TABLE 1:** Comparison of the mean scar scores according to the type of received treatment.

Time	Face (*n* = 20)		Between sides *p* ^a^	RM‐ANOVA		
	Laser treated side	Laser + isotretinoin treated side			*p*	Partial *η* ^2^
Baseline	7.95 ± 0.51	7.80 ± 0.52	0.379	Time	< 0.001^b^	0.875
3 months post‐treatment	6.50 ± 0.37	5.75 ± 0.39	< 0.001^b^	Side	< 0.001^b^	0.455
6 months post‐treatment	5.95 ± 0.32	4.70 ± 0.36	< 0.001^b^	Time × Side	0.003^b^	0.485
Within side *p*	< 0.001^b^	< 0.001^b^				

*Note:* The data are shown as Mean ± SE. *p*‐values are from a two‐way repeated‐measure ANOVA with two within factors (time, side).

Abbreviation: RM, repeated measure.

^a^ Pairwise comparisons were done by Bonferroni adjustment for multiple comparisons.

^b^ A *p*‐value less than 0.05 was considered statistically significant.

**FIGURE 2:**
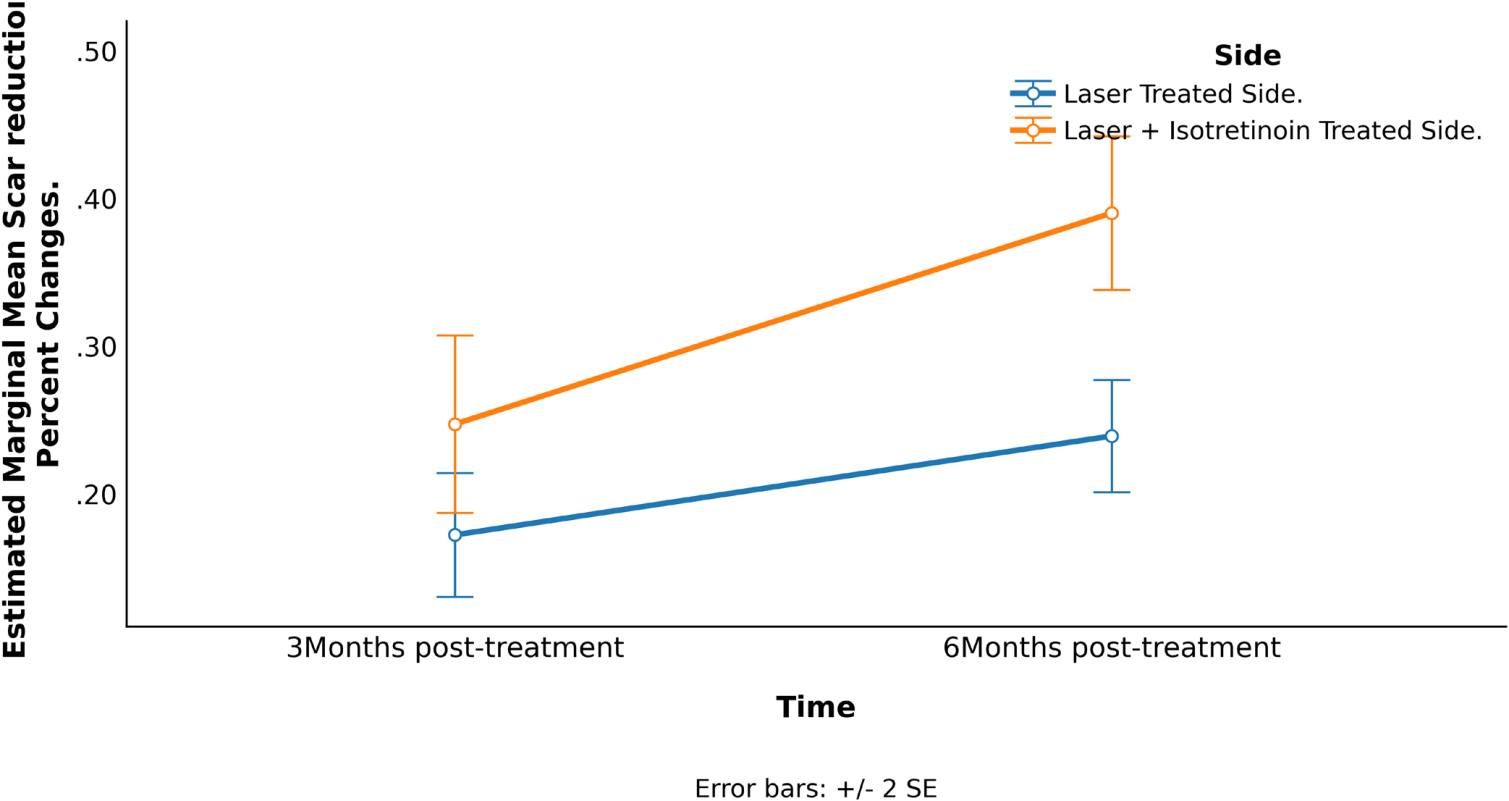
Scar reduction percent changes in both sides over study follow‐ups (mean ±2SE).

**FIGURE 3:**
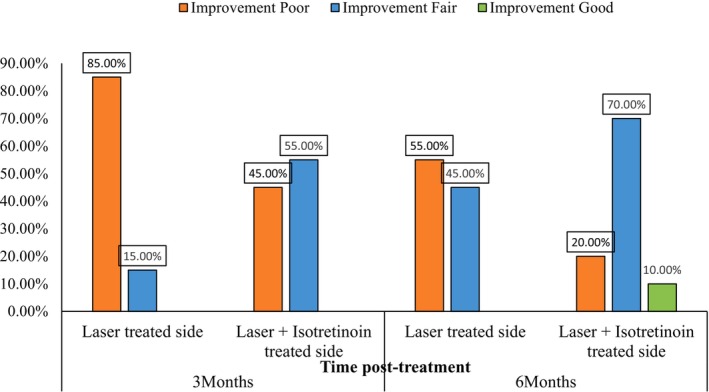
Patient scar improvement status based on the 3‐scale grading (≤ 25% = Poor, > 25% to ≤ 50 = Fair, > 50% = Good) at 3, 6 months post treatment.

Three months after treatment, we observed a significantly higher percentage of improvement in the scars on the laser + isotretinoin‐treated side compared to the laser‐treated side (24.7% ± 3.00% vs. 17.2% ± 2.10%; *p*‐value = 0.002). Additionally, the average improvement in the scars on the side treated with laser + isotretinoin was significantly greater than on the side treated with laser alone at 6 months after treatment (39.0 ± 2.60 vs. 23.9 ± 1.90; *p*‐value < 0.001) (Table 2). We observed that the scar reduction was considerably sharper in the laser + isotretinoin‐treated side than the laser‐treated side (Figure 2). As shown in the Figure 3, 3 months after treatment in the side that received laser, the most scars showed poor improvement (85%: ≤ 25% reduction), while in the side that received laser + isotretinoin, the most scars showed fair improvement (55%: > 25% to ≤ 50% reduction). Six months after treatment, on the side that received laser, only 45% of scars showed fair improvement, while on the side that received laser + isotretinoin, 70% of scars showed fair improvement (Figure 3).

**TABLE 2:** Comparison of the mean scar reduction percent change according to the type of received treatment.

Time	Face (*n* = 20)		Between sides *p* ^a^	RM‐ANOVA		
	Laser treated side	Laser + isotretinoin treated side			*p*	Partial *η* ^2^
3 months post‐treatment	17.2 ± 2.10	24.7 ± 3.00	0.002^b^	Time	< 0.001^b^	0.670
6 months post‐treatment	23.9 ± 1.90	39.0 ± 2.60	< 0.001^b^	Side	< 0.001^b^	0.573
Within side *p*	0.003^b^	< 0.001^b^		Time × Side	0.003^b^	0.375

*Note:* The data are shown as Mean ± SE. *p*‐values are from a two‐way repeated‐measure ANOVA with two within factors (time, side).

Abbreviation: RM, repeated measure.

^a^ Pairwise comparisons were done by Bonferroni adjustment for multiple comparisons.

^b^ A *p*‐value less than 0.05 was considered statistically significant.

### 3.3 Complications

No serious or persistent complications were observed at 3 and 6 months after treatment. Only one patient on the side that received laser + isotretinoin experienced erythema, which resolved spontaneously.

## 4 Discussion

The present study aimed to compare the combined effect of 1064 nm Q‐switched Nd:YAG laser and topical isotretinoin with 1064 nm Q‐switched Nd:YAG laser alone in the treatment of acne scars. The results demonstrated that the combination therapy provided significantly greater improvement in scar scores and percentage change at both 3‐ and 6‐month follow‐up compared to laser monotherapy, with no severe adverse effects. Only one case of transient erythema was observed, which resolved spontaneously.

Prior literature consistently documents the efficacy and safety of fractional 1064‐nm short‐pulse platforms for atrophic scarring. Clinical series and split‐face studies using fractional nanosecond Q‐switched 1064‐nm or fractional picosecond 1064‐nm systems report meaningful textural improvement with favorable tolerability, including in Asian skin types [13, 14, 18].

The enhanced efficacy observed with the addition of topical isotretinoin may be explained by several biological mechanisms. Retinoids are known to regulate keratinocyte differentiation, normalize follicular epithelial turnover, and show anti‐inflammatory effects through downregulation of Toll‐like receptor‐2 and pro‐inflammatory cytokines involved in acne pathogenesis. Other than their effects on active acne, retinoids stimulate dermal fibroblast activity and collagen synthesis while inhibiting matrix metalloproteinases, thereby promoting dermal remodeling. Fractional Q‐switched 1064‐nm Nd:YAG lasers induce controlled micro‐injury within the dermis through photoacoustic and photothermal effects, triggering neocollagenesis and elastin reorganization. The concurrent use of topical isotretinoin may get the dermal environment ready for enhanced wound healing and collagen deposition following laser‐induced injury. This biological synergy is supported by clinical studies demonstrating improved outcomes when fractional lasers are combined with isotretinoin, without increased risk of impaired wound healing or scarring [8, 15, 16, 19, 20].

Although ablative fractional CO_2_ often yields larger single‐course gains, comparative work shows this benefit comes with greater downtime and PIH risk, while Q‐switched 1064‐nm approaches provide a more conservative efficacy‐to‐safety trade‐off, an important consideration for higher phototypes [12].

Our results also align with emerging data that retinoids can be used concomitantly with fractional lasers without excess wound‐healing complications and may enhance outcomes. Randomized and prospective studies have demonstrated the safety and efficacy of pairing fractional lasers with low‐dose isotretinoin—including concurrent protocols—across acne and early scar indications [10, 15, 16].

A large systematic review further concluded that the traditional 6–12‐month delay after isotretinoin lacks firm evidence and that many procedures, including lasers, can be performed safely with appropriate patient selection [19]. Additionally, observational data suggest patients on isotretinoin may experience greater improvements with 1550‐nm fractional treatment, supporting a biological synergy between retinoid‐induced dermal remodeling and laser‐induced neocollagenesis [21].

Mechanistically, fractional short‐pulse 1064‐nm devices create microscopic intradermal injury zones as it has photothermal and photoacoustic effects. This stimulates neocollagenesis while sparing surrounding tissue; histological studies from fractional 1064‐nm picosecond treatments demonstrate increased dermal remodeling consistent with these effects [20]. Reviews of acne‐scar management further place Q‐switched/ps 1064‐nm lasers within the broader energy‐based armamentarium, emphasizing device selection by skin type, downtime tolerance, and scar morphology [6].

Strengths of our study include the randomized, assessor‐blinded split‐face design and standardized laser parameters, which reduce inter‐individual variability. Limitations include the modest sample size and 6‐month horizon, which may not capture durability of effect or rare late events. A limitation of this study is the lack of stratified analysis based on acne scar morphology (e.g., ice‐pick, boxcar, rolling scars). Although fractional lasers may exert differential effects depending on scar type, the current study was not powered to support robust subtype‐specific statistical comparisons. Future studies with larger sample sizes and pre‐defined stratification by scar morphology are warranted to determine whether combination therapy offers preferential benefit for specific scar subtypes. The present study was not powered to assess sex‐based differences in treatment response, particularly because only four male patients were included. Although no clear sex‐specific pattern of response or adverse effects was observed clinically, larger studies with more balanced sex distribution are needed to determine whether treatment outcomes differ between male and female patients.

Future trials should include longer follow‐up, stratification by scar subtype, and dose–response optimization for topical isotretinoin. Our findings nonetheless add controlled evidence that combining topical isotretinoin with 1064‐nm QSF Nd:YAG can enhance clinical improvement over laser monotherapy without compromising safety, complementing reports using oral isotretinoin with fractional systems.

Clinical implications: For patients seeking meaningful scar improvement with low downtime and PIH risk, a course of fractional 1064‐nm short‐pulse laser can be positioned as a skin‐type‐inclusive option, and adjunct topical isotretinoin may further potentiate outcomes under careful monitoring—particularly when strict photoprotection and gentle skin care are enforced. Comparative trials against ablative fractional CO_2_/Er:YAG will clarify relative effect sizes, but existing data suggest a favorable efficacy‐to‐tolerability balance for 1064‐nm short‐pulse platforms in appropriate candidates.

## 5 Conclusion

Acne scars are a common sequela that affect the patient's appearance and subsequently the psychological state of the individual. The combination of topical isotretinoin and Fractional Q‐Switched 1064 nm laser is more effective than laser alone in improving acne scars, without significant side effects.

## Funding

The authors have nothing to report.

## Ethics Statement

This study was approved by the Ethical Committee of Isfahan University of Medical Sciences (resolution code: IR.MUI.MED.REC.1402.404).

## Conflicts of Interest

The authors declare no conflicts of interest.

## Data Availability

The data that support the findings of this study are available on request from the corresponding author. The data are not publicly available due to privacy or ethical restrictions.
